# A Versatile Macromer-Based Glycosaminoglycan (sHA3) Decorated Biomaterial for Pro-Osteogenic Scavenging of Wnt Antagonists

**DOI:** 10.3390/pharmaceutics12111037

**Published:** 2020-10-29

**Authors:** Mathis Gronbach, Franziska Mitrach, Stephanie Möller, Sandra Rother, Sabrina Friebe, Stefan G. Mayr, Matthias Schnabelrauch, Vera Hintze, Michael C. Hacker, Michaela Schulz-Siegmund

**Affiliations:** 1 Pharmaceutical Technology, Medical Faculty, University of Leipzig, Eilenburger Str. 15A, 04317 Leipzig, Germany; mathis.gronbach@uni-leipzig.de (M.G.); franziska.mitrach@uni-leipzig.de (F.M.); michael.hacker@hhu.de (M.C.H.); 2 Biomaterials Department, INNOVENT e.V., Pruessingstraße 27B, 07745 Jena, Germany; sm@innovent-jena.de (S.M.); ms@innovent-jena.de (M.S.); 3 Max Bergmann Center of Biomaterials, Technische Universität Dresden, Budapester Str. 27, 01062 Dresden, Germany; sandra.rother@tu-dresden.de (S.R.); vera.hintze@tu-dresden.de (V.H.); 4 Department of Cellular and Molecular Medicine, Glycobiology Research and Training Center, University of California, San Diego, La Jolla, CA 92093-0687, USA; 5 Leibniz-Institut für Oberflächenmodifizierung e.V. (IOM), Permoserstr. 15, 04318 Leipzig, Germany; sabrina.friebe@iom-leipzig.de (S.F.); stefan.mayr@iom-leipzig.de (S.G.M.); 6 Division of Surface Physics, University of Leipzig, Linnéstraße. 5, 04103 Leipzig, Germany; 7 Institute of Pharmaceutics and Biopharmaceutics, Heinrich-Heine University, Universitätsstr. 1, 40225 Düsseldorf, Germany

**Keywords:** hyaluronan, sclerostin, DKK1, surface modification, tissue engineering, scavenging, bone regeneration, Wnt-signaling, biodegradable polymer

## Abstract

High serum levels of Wnt antagonists are known to be involved in delayed bone defect healing. Pharmaceutically active implant materials that can modulate the micromilieu of bone defects with regard to Wnt antagonists are therefore considered promising to support defect regeneration. In this study, we show the versatility of a macromer based biomaterial platform to systematically optimize covalent surface decoration with high-sulfated glycosaminoglycans (sHA3) for efficient scavenging of Wnt antagonist sclerostin. Film surfaces representing scaffold implants were cross-copolymerized from three-armed biodegradable macromers and glycidylmethacrylate and covalently decorated with various polyetheramine linkers. The impact of linker properties (size, branching) and density on sHA3 functionalization efficiency and scavenging capacities for sclerostin was tested. The copolymerized 2D system allowed for finding an optimal, cytocompatible formulation for sHA3 functionalization. On these optimized sHA3 decorated films, we showed efficient scavenging of Wnt antagonists DKK1 and sclerostin, whereas Wnt agonist Wnt3a remained in the medium of differentiating SaOS-2 and hMSC. Consequently, qualitative and quantitative analysis of hydroxyapatite staining as a measure for osteogenic differentiation revealed superior mineralization on sHA3 materials. In conclusion, we showed how our versatile material platform enables us to efficiently scavenge and inactivate Wnt antagonists from the osteogenic micromilieu. We consider this a promising approach to reduce the negative effects of Wnt antagonists in regeneration of bone defects via sHA3 decorated macromer based macroporous implants.

## 1. Introduction

In regenerative medicine the ability to easily vary characteristics of applied materials in a well-defined manner is of great importance [1]. Biomaterials made of crosslinkable oligomeric macromers provide the demanded flexibility via copolymerization of different components [2] allowing for variable crosslinking degrees and properties, such as mechanical strength, biodegradability, and lipophilicity. Surface modification offers an opportunity to add further pharmaceutically beneficial functionalities to the material-inherent properties of e.g. scaffolds [3]. In a clinical context the modification may enhance material integration and tissue regeneration [4]. Linker molecules carrying accessible functional groups enable a controlled binding of bioactive molecules and thereby pharmaceutical functionalization [5]. Widely discussed linker properties are length and density on the surface, which are decisive for an effective presentation of functionalizing molecules to the materials surroundings [6,7]. A local application at defined defect sites is a main advantage in comparison to systemically administered active ingredients.

For the analysis of bone-regenerative materials, it is therefore important to provide an approach suitable to analyze and quantify surface linker-modification and finally functionalization. In a recent study, we established a general protocol for surface analysis based on the processing of oligomeric macromers into films [8]. We employed a three-armed biodegradable macromer as a cross-polymerizable building block in a film formulation that corresponds to the surface properties of porous 3D scaffolds already applied in vivo [9].

Cross-copolymerization with a functionalized linker, however, relies on the reactivity of the linker with the macromer as well as on suitable solvent systems [8]. In order to master these challenges and provide more flexibility, our approach is to incorporate a small anchor molecule, glycidyl methacrylate (GMA), into the film, and add in a second step an adaptable linker molecule to the material surface. The film platform was adjusted to the needs of this modified approach and a library of commercially available polyetheramines was tested. This variability represents a main advantage of the system presented here, as it allows to test different linkers of varying properties (such as branching, chain length, and hydrophilicity), while still maintaining standardized polymerization conditions in the first place. The influence of the linker itself and the amount of surface decoration were tested regarding the ability to modify and eventually functionalize the films derived from the material.

As the main macromer was already applied in vivo for its bone regenerative properties [9,10], a model functionalization related with this approach, namely high-sulfated hyaluronan (sHA3), was chosen. Sulfated glycosaminoglycans, in general, are known for their bone-regenerative potential, as they have been shown to support osteoblast [11] and suppress osteoclast functions [12]. In a prior study, we could demonstrate the feasibility of this concept [13]. As a basis for the results presented here, the ability of sHA3 functionalized surfaces to scavenge Dickkopf-1 (DKK1), resulting in a pro-osteogenic effect was shown. Other studies suggest a strong interaction with sclerostin (SOST) may be another mechanism how sulfated hyaluronans beneficially impact bone regeneration [14]. Here, we therefore aim to enable and optimize the covalent functionalization of our macromer surfaces with sHA3 in order to scavenge and thereby inactivate both, DKK1 and sclerostin from the bone micro-milieu and introduce a promising biomaterial approach for bone regeneration [15].

Employing our analytical film platform, we investigated polyetheramine linker molecules of different size and valence, suitable functionalization strategies and the necessary anchor concentration for effective scavenging of the Wnt (Wingless-related integration site) antagonists. In addition, we analyzed the affinity of sHA3 towards our target protein sclerostin via SPR measurements.

In a final step, we examined the ability of sHA3 functionalized macromer films to scavenge clinically relevant amounts of sclerostin [16,17] among serum proteins as well as in a competitive binding scenario with DKK1 and the pro-osteogenic Wnt ligand Wnt3a. The relevance of this scenario was investigated in cell culture experiments with hMSC (human mesenchymal stem cells) and SaOS-2 cells upon osteogenic differentiation.

The knowledge about a controlled surface modification and functionalization acquired using the variable two-step system presented here will be helpful when extrapolating the results to 3D scaffolds [10] and surface modification of already established implant materials with potential clinical application.

## 2. Materials and Methods

### 2.1. Materials

All glycosaminoglycans (GAGs) applied in this study were synthesized and characterized according to established protocols [18] at Innovent e.V., Jena. High-sulfated glycosaminoglycan sHA3 had the following characteristics: MW = 74.600, degree of sulfation = 3.4 and an ATTO-565 labeling. In addition, unlabeled GAG (degree of sulfation: 3.4) and an unsulfated hyaluronan (HA) were applied in the SPR (surface plasmon resonance) measurements.

Huntsman Chemical (The Woodlands, TX, USA) generously provided polyetheramines Jeffamine^®^ EDR148, ED600, ED900, ED2003, T403, and T3000. The nomenclature describes the branching of the polyetheramine with “ED” referring to two-armed and “T” to three-armed Jeffamines^®^. The following number stands for the molecular weight of the respective polyetheramine.

All other chemicals (if not stated differently) were purchased from Sigma-Aldrich/Merck (Darmstadt, Germany).

### 2.2. Fabrication of Polymer Films

Macromers applied were synthesized as described before [19]. The three-armed macromer T134LA6 was used in this study. The nomenclature is referring to its trimethylolpropane core, which has a molecular weight of 134. In addition, each arm carries six lactic acids, ending with a methacrylate capping, in order to allow for radical polymerization reactions. A previously described technical setup [8] was used to produce cross-polymerized copolymer films.

Briefly, a thin film consisting of T134LA6 and a variable amount of the reactive anchor molecule glycidyl methacrylate (GMA) (Supplementary Material, Figure S1) was cross-copolymerized between covalent carrier glass disks (diameter: 2 cm) and object slides.

Glass carriers were silanized using 3-(trimethoxysilyl)propylmethacrylate, whereas object slides were treated with 2-(carbomethoxy) ethylmethyldimethoxysilane, following a previously described protocol [20]. In an additional cleaning step, glass carriers were exposed to oxygen plasma (flow rate of 10 standard cubic centimeters per minute O_2_, 0.2 mbar, and 100 W) for 2 min prior to silanization.

As solvent, a previously established 5:3 (volume parts) mixture of acetone and dichloromethane (binary) and different single-solvent approaches (dioxane, ethyl acetate, and tetrahydrofuran) were applied. In addition, varying durations (1–5 h) and temperatures ranging from 40 to 65 °C were tested during the establishment of the polymerization process. The polymerization took place under nitrogen atmosphere.

### 2.3. Modification of the Film Surface—Introduction of a Linker Molecule

In order to be able to perform further functionalization of the surfaces a reaction to bind the linker molecule is necessary. This was carried out using 0.055 M of the respective polyetheramine (Supplementary Material, Figure S1) and 0.1% (*m*/*m*) 2,4,6-tris(dimethylaminomethyl)phenol (DMP30) as an accelerator in *N*,*N*-dimethylformamide (DMF) as a modification mixture. Different durations and temperatures were tested and adapted for each polyetheramine linker. To verify that the polyetheramine reacts with the epoxy group of the GMA anchor and not the macromer itself, films without incorporated GMA were incubated under the same conditions. Subsequently, films were washed in tetrahydrofuran (THF) for 20 min, dried, and stored under vacuum.

In order to reduce the materials possible ionic interaction, acetylated surfaces were generated [13]. Amino modified surfaces were exposed to acetic anhydride and trimethylamine (2:3 *v*/*v*) in THF.

### 2.4. Analysis of the Surface Decoration

In order to be able to detect and quantify linker decoration on the film surface 6-(fluorescein-5-(and-6)-carboxamido) hexanoic acid, succinimidyl ester (5(6)-SFX) was used, a fluorescent dye which specifically reacts with free primary amino groups. The binding was conducted using a 0.256 mM 5(6)-SFX solution in 0.1M phosphate buffer at a pH value of 6.95. Staining solution was added to each film in a 12-well plate and incubated overnight at 8 °C while constant shaking at 40 rpm. Subsequently, films were washed two times with deionized water. For quantification, a GE Typhoon 9410 Imager (GE Healthcare, Freiburg, Germany) high resolution fluorescence scanner (excitation: 488 nm, emission: 520 nm) was used.

### 2.5. Functionalization of the Films

Labeling of sHA3 (Supplementary Material, Figure S1) with the fluorescent dye ATTO-565 allowed quantification via the high-resolution Typhoon fluorescence scanner.

The functionalization of the films with respective GAG was performed using a concentration of 0.1 M 1-ethyl-3-(-3-dimethylaminopropyl) carbodiimide hydrochloride (EDC) and 0.05 M *N*-hydroxysuccinimide (NHS) in the mentioned phosphate buffer.

After addition of the sHA3 (0.37 mg/mL) to the mixture, coupling was carried out overnight at room temperature and under reduced light exposure while constant shaking at 40 rpm.

In order to determine the difference between adsorptively and covalently bound GAGs, functionalized films and controls were washed in different buffer systems for 30 min under constant stirring.

### 2.6. Surface Plasmon Resonance (SPR) Interaction Analysis

Binding affinity of HA derivatives for sclerostin was analyzed using a Biacore T200 instrument (GE Healthcare, Freiburg, Germany) with HBS-EP buffer (0.01 M HEPES, 0.15 M NaCl, 3 mM EDTA, 0.05% (*v*/*v*) surfactant P20, pH 7.4) at 37 °C. Recombinant human Sclerostin (R&D Systems, Minneapolis, MN, USA) was immobilized onto Series S Sensor Chips CM5 via amine coupling at 25 °C as described by GE Healthcare, resulting in immobilization values of about 2150 RU. As a reference, an activated, and afterwards deactivated, flow cell without immobilized protein was used. GAGs were injected for 300 s at concentrations of 50 and 500 µM related to the molecular weight of their disaccharide units (D.U.) followed by a dissociation phase of 800 s. A total of 10 s before the end of sample injection respective binding levels (relative to the baseline) were detected. For chip surface regeneration after each measurement, a 60 s injection of 5 M NaCl and 30 mM NaOH, followed by a stabilization period of 1000 s in running buffer was applied. Binding parameters were evaluated using the Biacore evaluation software 2.03 (GE Healthcare, Freiburg, Germany, 2019). Sensorgrams were obtained by double referencing.

### 2.7. Scavenging Studies

All scavenging studies were performed in suspension cell culture plastics (12-well suspension plate, Sarstedt, Nuembrecht, Germany) in order to avoid an interaction of proteins with the material. The concentration of recombinant human sclerostin (R&D Systems, Minneapolis, MN, USA) used during these scavenging experiments was 8 ng/mL. First studies were conducted by incubation of the films in a buffer system. Bovine serum albumin (0.1%) and Polysorbate 80 (0.2%) were added in order to stabilize the phosphate-buffered saline (PBS) solution. Materials were incubated in this solution under cell culture conditions (37 °C, 5% CO_2_) over a period of 24 h while constantly shaking. Cell culture medium (DMEM supplemented with 10% of fetal bovine serum (FBS)) was applied during all further approaches. Samples were incubated with recombinant human Wnt3a alone (R&D Systems, Minneapolis, MN, USA) or in combination with human, recombinant sclerostin and DKK1 (R&D Systems, Minneapolis, MN, USA) in a 1:1 molar ratio applying the same conditions.

Supernatants were collected and unbound protein was quantified via human sclerostin, human DKK1 DuoSet^®^ Enzyme-linked immunosorbent assay (R&D Systems, Minneapolis, MN, USA) and Wnt3a ELISA Kit (Bioassay Technology Laboratory, Shanghai, China).

### 2.8. Binding Stability and Scavenging Capacity

In order to test for the binding strength of sHA3 towards sclerostin over time, films were incubated with a surplus (40 ng/mL) of protein overnight. During the subsequent incubation of the presaturated samples under cell-culture conditions, ELISA measurements were performed at every medium change (days 1, 3, 6, 8, and 10) and results cumulated.

### 2.9. Varying GMA Concentrations

The influence of increasing amounts of GMA (5%, 10%, 20%, and 30%, 40% *m*/*m*) incorporated during the cross-copolymerization was observed. All formulations were tested regarding their ability towards linker modification, sHA3 decoration, and the impact on scavenging capabilities, applying the established methods.

### 2.10. Cytocompatibility Assay

L929 fibroblasts (Cell Lines Service, Eppelheim, Germany) were applied to test for cytotoxicity of our materials. After seeding, cells were kept under cell-culture conditions for 18 h in order to assure attachment to the respective material surface. Cell viability was measured using ROTITEST^®^ Vital (Carl Roth GmBH & Co. KG, Karlsruhe, Germany) according to the manufacturer’s instructions. The viability of the cells exposed to various surfaces was calculated as percentage to the control (cells seeded on tissue culture polystyrene (TCPS)).

### 2.11. Cell Culture 

SaOS-2 cells were cultured in standardized medium (DMEM, low glucose supplemented with 10% FBS and 1% penicillin/streptomycin) for 24 h. Afterwards osteogenic differentiation was initiated by changing to osteogenic medium (OM), consisting of DMEM, low glucose supplemented with 10% FBS and 1% penicillin/streptomycin, 100 nM dexamethasone, 285 µM ascorbic acid, and 10 mM β-glycerophosphate. Human MSC from Lonza (Basel, Switzerland) were derived from bone marrow aspirates of a 22-year-old healthy, Caucasian male. The cells (hMSC) were treated in the same manner except for the addition of 1% nonessential amino acids to both, growth and osteogenic medium.

Cell supernatants were collected on every medium change and respective sclerostin, DKK1 and Wnt3a concentrations determined using ELISA.

### 2.12. Osteogenic Differentiation

Calcium AS FS (DiaSys Diagnostic Systems, Holzheim, Germany) was used to quantify calcium content (normalized to the cell number per well) after cell lysis. OsteoImage^TM^ mineralization assay (Lonza, Basel, Switzerland) applied according to the manufacturer’s instructions, allowed for hydroxyapatite visualization and quantification via the high resolution fluorescence scanner. Cells were stained with Alexa Fluor 568 phalloidin and examined using a Leica SP5X laser scanning confocal microscope (Leica, Wetzlar, Germany) for qualitative assessment. Leica Applications Suite was used to obtain images.

### 2.13. Statistical Analysis

All experiments were performed in at least triplicates. Data was analyzed using GraphPad Prism V. 6.01 (GraphPad Software, La Jolla, CA, USA). Statistical significance was determined by one-way ANOVA with Tukey post-hoc test. Results are given as the mean ± standard deviation (SD), and *p* values below 0.05 were considered as statistically significant.

## 3. Results

### 3.1. Film Manufacturing and Linker Modification

#### 3.1.1. Optimized Polymerization Conditions for Model Films

Our first step was to transfer the copolymerization of T134LA6 and GMA to our surface model concept. Varying polymerization and incubation conditions were tested in order to establish a protocol allowing for a reproducible film manufacturing process.

Regarding temperature and duration of the cross-copolymerization, a protocol involving 2 h at 65 °C resulted in fully hardened films grafted onto the glass disks and therefore was applied for all following experiments. Lower temperature led to an incomplete polymerization, characterized by inhomogeneous films. A prolonged time span showed no improvement.

**Figure 1:**
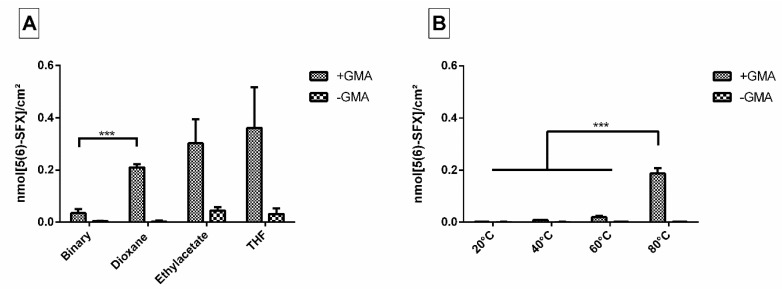
(**A**) Immobilized 5(6)-SFX indicating the amount of accessible, derivatizable amino groups introduced to the film surface via ED900 linker addition after cross-copolymerization with and without GMA using different solvents. Binary solvent mixture consisted of acetone and DCM (5:3). (**B**) 5(6)-SFX bound to film surfaces with and without GMA after incubation at different temperatures. The applied cross-copolymerization solvent is dioxane, short incubation periods (2 h) at 80 °C were compared to longer incubation (15 h) at 20, 40, and 60 °C. All values represent the mean ± SD of *n* ≥ 4. *** *p* < 0.001 vs. respective treatment.

When we applied the binary solvent system as established in the former study [8], we found films with even surfaces, but detected only low amounts of free amino groups on their surface after incubation with ED900 as a linker (Figure 1A). As the anchor GMA provides a good solubility in several organic solvents even in the presence of a growing polymer network, we were able to test single solvent systems.

Within the different single solvent approaches tested, dioxane showed the best results regarding smooth and homogeneously amino-functionalized films.

#### 3.1.2. Effect of Different Modification Temperatures on Polyetheramine Binding

In order to effectively bind polyetheramine as a hydrophilic linker to the film surface, we had to determine suitable reaction conditions. As a measure of efficiency, we quantified the amount of free amino groups introduced to the material surface via binding of ED900 and subsequent 5(6)-SFX staining and analysis with a high-resolution fluorescence scanner.

First, different reaction temperatures were applied. Low temperatures resulted in only small amounts of amine groups (0.001 nmol/cm^2^ even after 15 h at 40 °C) with no significant difference to the control without the GMA anchor incorporated (Figure 1B). In contrast, a high temperature approach (80 °C) combined with a relatively short incubation time of 2 h showed promising conversion rates and was therefore chosen for further experiments.

#### 3.1.3. Variation of Linker and the Incubation Time

In a next step, we tested two-armed linkers with a molecular weight in the range of 148–2003 g/mol and three-armed polyetheramine linkers with a molecular weight of 403 and 3000 g/mol. For each of these linker molecules, an optimized incubation duration was found by determination of a time point with a maximum of directed linker decoration. Prolonged incubation durations resulted in undirected linker addition, characterized by an increasing amount of 5-(6)SFX detected on the control group without GMA anchor incorporated.

**Figure 2:**
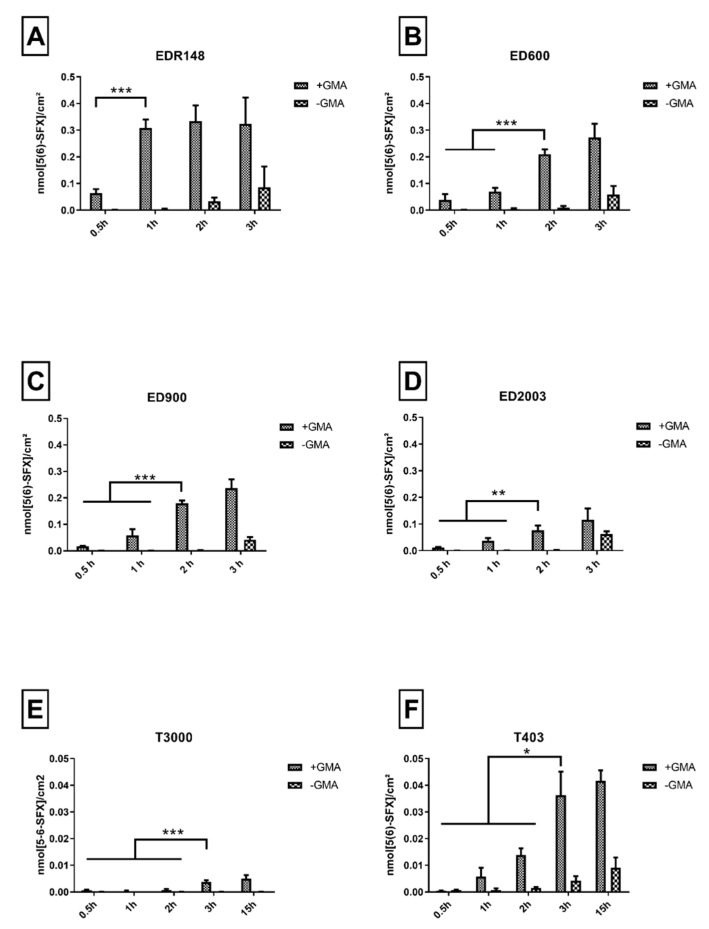
Immobilized 5(6)-SFX indicating the amount of accessible, derivatizable amino groups on the film surface. Values measured on the film surface after cross-copolymerization with and without GMA and subsequent incubation with different linker molecules over varying periods. (**A**–**D**) represent the results for two-armed, (**E**,**F**) for three-armed polyetheramine-based linker molecules applied. All values represent the mean ± SD of *n* ≥ 4. * *p* < 0.05; ** *p* < 0.01; *** *p* < 0.001 denotes significant difference vs. the respective treatment.

EDR148 showed fast reaction kinetics with values of 0.31 nmol/cm^2^ reached after 1 h of incubation (Figure 2A). No significant increase of 5-(6)SFX binding was detected for later time points, while an increasing amount of amino groups detected on the non GMA containing materials could be found after 2 and 3 h. Overall, the highest density of free amino groups available for further functionalization in comparison to all tested linkers was detected for this small linker. With increasing molecular weight of the linker, we observed an increase in grafting time and a decrease in grafting efficiency that resulted in about 0.2 nmol/cm^2^ for ED600 (Figure 2B) and ED900 (Figure 2C) and only 0.08 nmol/cm^2^ for ED2003 (Figure 2D). Concomitantly, unspecific binding to GMA-free macromer films increased with time and limited effective binding to a 2 h reaction time.

Incubation with the three-armed polyetheramines, i.e., T403 (0.014 nmol/cm^2^, Figure 2E) and T3000 (0.004 nmol/cm^2^, Figure 2F) resulted in strongly reduced amino group density on the surface available for 5-(6)SFX binding. We therefore dismissed the three-armed linkers for further experiments.

In order to assure a sufficient and controllable linker modification, while avoiding undirected amino functionalization, a 2 h incubation for ED600 and ED900 and a 1-h period for EDR148 at 80 °C were chosen for further experiments.

### 3.2. Surface Functionalization

#### 3.2.1. SPR Demonstrates Binding Strength of sHA3 Used for Functionalization towards Sclerostin

**Figure 3:**
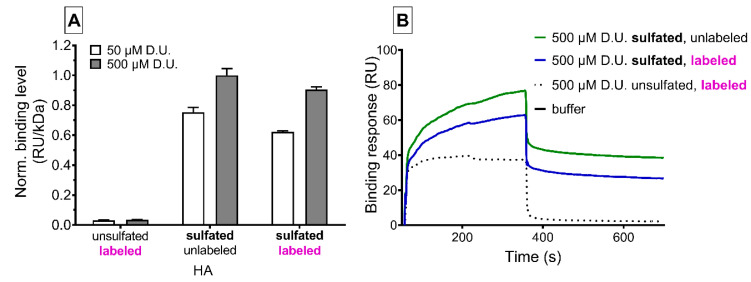
Surface plasmon resonance analysis of sclerostin/GAG interaction, reported as resonance units (RU). (**A**) Relative binding levels of labeled and unlabeled sulfated or native hyaluronan and corresponding sensorgrams (**B**). GAGs were injected at 50 and 500 µM related to the molecular weight of their disaccharide units (D.U.).

Surface plasmon resonance interaction studies showed the affinity of GAG variants to the Wnt inhibitor sclerostin (Figure 3). We found a sulfation- and concentration-dependent interaction of sHA3 derivatives with or without ATTO-labeling to sclerostin decorated chip surfaces compared to nonsulfated ATTO-labeled GAGs. Labeling of sHA3 with the dye slightly reduced the binding response. After GAG injection, nonsulfated HA quickly dissociated from sclerostin while both sHA3 derivatives remained partially bound to sclerostin.

#### 3.2.2. Establishing a Washing Protocol to Remove Adsorptively Bound GAG

**Figure 4:**
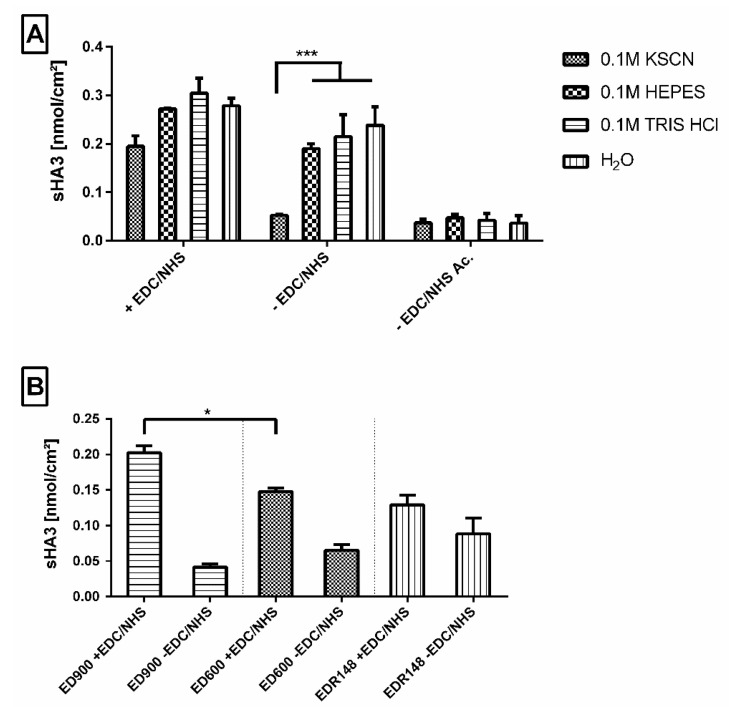
(**A**) Effect of different washing buffers on the amount of fluorescence labeled sHA3 detected on ED900 modified film surfaces. Sulfated hyaluronan was introduced with (+EDC/NHS) and without (-EDC/NHS) the addition of EDC/NHS reagent mix to ED900 modified surfaces and to ED900 acetylated (-EDC/NHS Ac.) control groups. (**B**) Effect of different linkers on the amount of fluorescence labeled sHA3 detected on modified film surfaces. Sulfated hyaluronan was introduced with (+EDC/NHS) and without (-EDC/NHS) the addition of EDC/NHS reagent to polyetheramine EDR148, ED600, and ED900 modified surfaces. All values represent the mean ± SD of *n* ≥ 4. * *p* < 0.05, *** *p* < 0.001 denotes significant difference vs. the respective surface modification.

Our macromer film surface design with free amino groups allowed for direct sHA3 binding via EDC/NHS chemistry. For ED900 modified films, we were able to bind and detect an amount of sHA3 comparable to the number of available linker molecules determined earlier by 5(6)-SFX binding. We therefore used these films to find a suitable washing protocol to remove adsorptively bound sHA3, visible on films incubated with sHA3 but without EDC/NHS treatment (Figure 4A). We employed several different buffers, i.e., KSCN, 4-(2-hydroxyethyl)-1-piperazineethanesulfonic acid (HEPES) and tris(hydroxymethyl)aminomethane (TRIS) buffer and investigated the remaining sHA3 on the film surface. KSCN buffer with a concentration of 0.1 mM (pH = 6.95) showed the best results in reducing adsorptively bound sHA3 to a total amount of 0.042 nmol/cm^2^ and therefore was used as a standard washing protocol for the upcoming experiments.

Acetylated control surfaces were generated in order to mitigate ionic interactions on adsorptive binding. Figure 4A shows reduced binding independent of the employed washing protocol and proves the assumed relevance of ionic interaction for adsorptive immobilization of sHA3.

#### 3.2.3. Impact of the Linker Molecule on Surface Functionalization

We compared the efficiency of sHA3 immobilization on films modified with ED148, ED600, and ED900. Modification with the fast-reacting EDR148 resulted in amounts of 0.12 nmol/cm^2^ sHA3 immobilized on the surface after incubation with EDC/NHS and washing in KSCN buffer (Figure 4B). With reference to ED600 and 900 modified films, the highest amount of adsorptively attached sHA3 was detected on ED148 films, resulting in nonsignificant differences between the activated and nonactivated amine surfaces.

In contrast to EDR148 films, for ED600 and ED900, significant differences between covalently and adsorptively bound sHA3 on the film surfaces were found. Most efficient sHA3 decoration was found for ED900, resulting in values of 0.20 nmol/cm^2^.

In general, a functionalization of all tested linker-modified materials was possible. The results for ED900 suggest a superior combination of accessibility towards covalent functionalization and the option to wash off adsorptively bound sHA3 in a sufficient way. We therefore decided to apply ED900 in all following experiments.

#### 3.2.4. Sclerostin Scavenging

**Figure 5:**
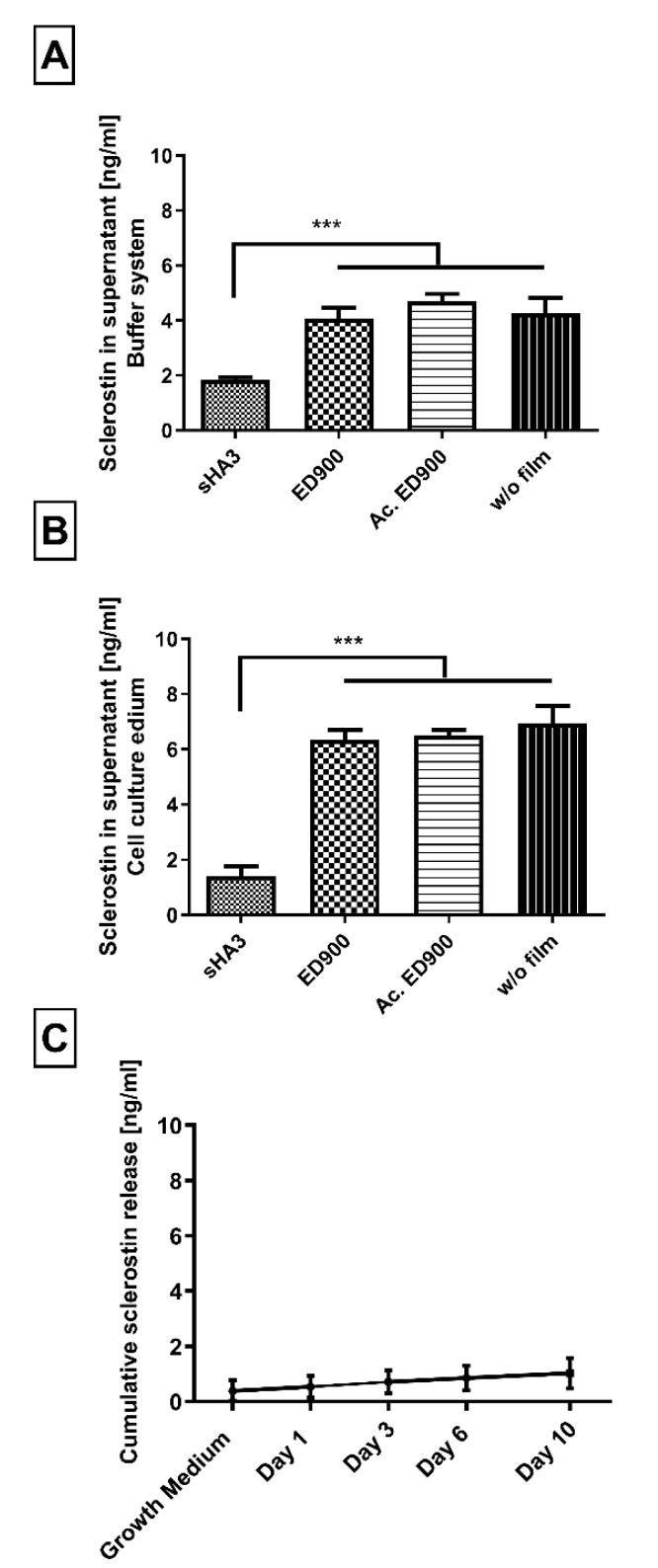
Sclerostin binding to variously decorated material films. (**A**–**C**) Differently modified surfaces were incubated with 8 ng/mL sclerostin. Binding was quantified by the amount of unbound sclerostin in the supernatant after 24 h (**A**,**B**). (**C**) Cumulative sclerostin release from a saturated and preincubated (40 ng/mL) sHA3 surface. Sclerostin was presented in either 0.1% BSA and 0.2% Tween 80 in PBS (**A**) or DMEM with 10% serum (**B**,**C**). Film surfaces: sHA3: covalent ED900 and sHA3-coating; ED900: ED900 modified film preincubated with soluble sHA3, Ac. ED900: Acetylated ED900 acetylated to neutralize positive charge; T134LA6: nonmodified film. All values represent the mean ± SD of *n* ≥ 4. *** *p* < 0.001 denotes significant difference vs. the respective surface modification.

sHA3 decorated ED900 macromer films were investigated for their sclerostin scavenging capability in model incubation systems. The incubation of sHA3 films and controls with a reasonable amount of sclerostin in a buffer solution led to the desired depletion of the supernatant over the functionalized material after 24 h. Recovery of sclerostin in control wells depended on the experimental set up (Figure 5A,B) and was optimized in cell culture medium with 10% FBS where almost all applied sclerostin was recovered (Figure 5B).

When we incubated sHA3 films with a surplus of sclerostin, in order to determine their scavenging capacity, we found 11.5 ± 0.4 ng/mL of scavenged sclerostin per cm^2^ functionalized film surface.

Incubation of sHA3 films presaturated with sclerostin led to no significant release of the Wnt-antagonist in the observed timeframe of 10 days (Figure 5C).

#### 3.2.5. Coincubation of Wnt3a, Sclerostin and DKK1

**Figure 6:**
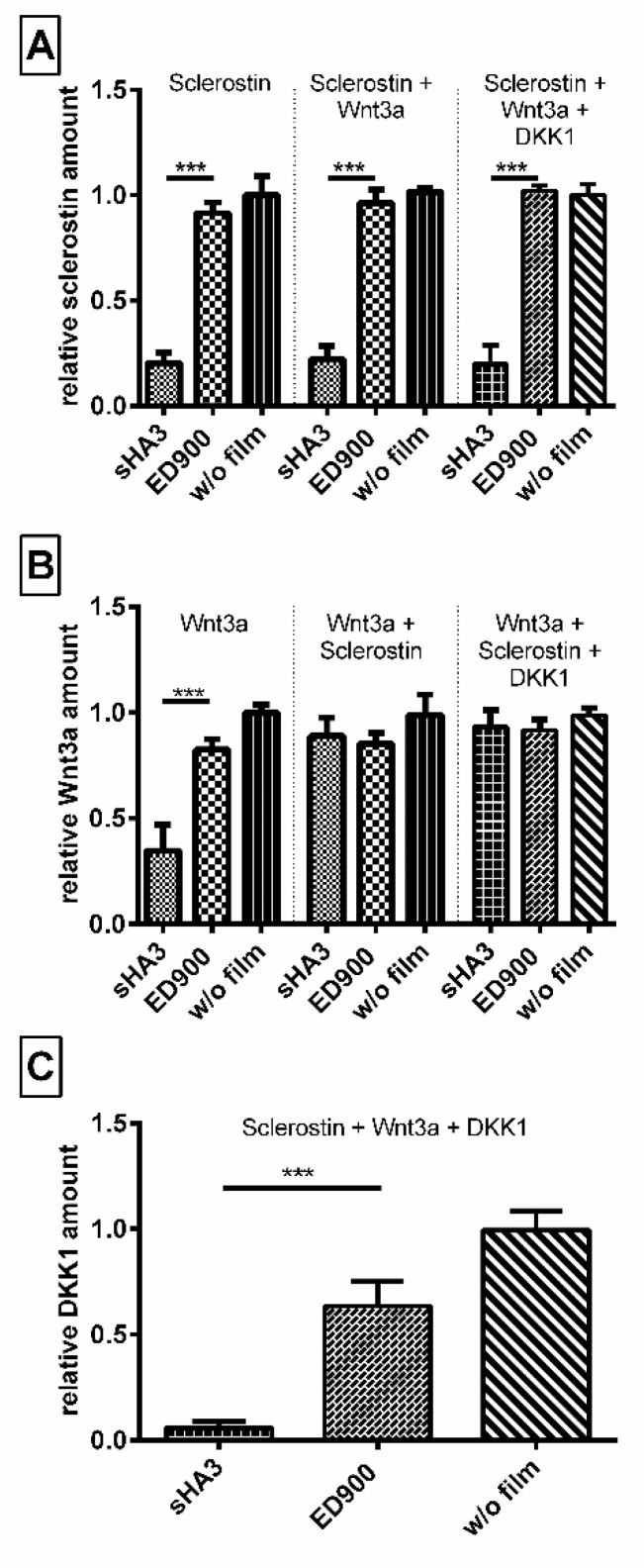
Sclerostin and DKK1 are the preferred binding partner of sHA3 functionalized films. Differently modified films were incubated with the same molar amounts of Sclerostin, Wnt3a, or both proteins combined. Unbound (**A**) Sclerostin, (**B**) Wnt3a, and (**C**) DKK1 was quantified after 24 h from the supernatant via ELISA. Recovery normalized to controls without any films (blank well plates). Film surfaces: ED900: ED900 incubated with soluble sHA3; sHA3: ED900 covalently coated with sHA3. All values represent the mean ± SD of *n* ≥ 4. *** *p* < 0.001 denotes significant difference vs. the respective surface modification.

In order to generate a competitive binding scenario equal molar amounts of sclerostin, DKK1 and Wnt3a, in combination and each on their own were incubated with our material surfaces (Figure 6). While we were able to prove an affinity of sHA3 towards Wnt3a, the coincubation reveals that in presence of either DKK1 or sclerostin and even all three proteins, the Wnt-antagonists sclerostin and DKK1 are the preferred binding partners of sHA3 functionalized surfaces.

#### 3.2.6. Impact of the Amount of Anchor Molecule Incorporated during Polymerization

**Figure 7:**
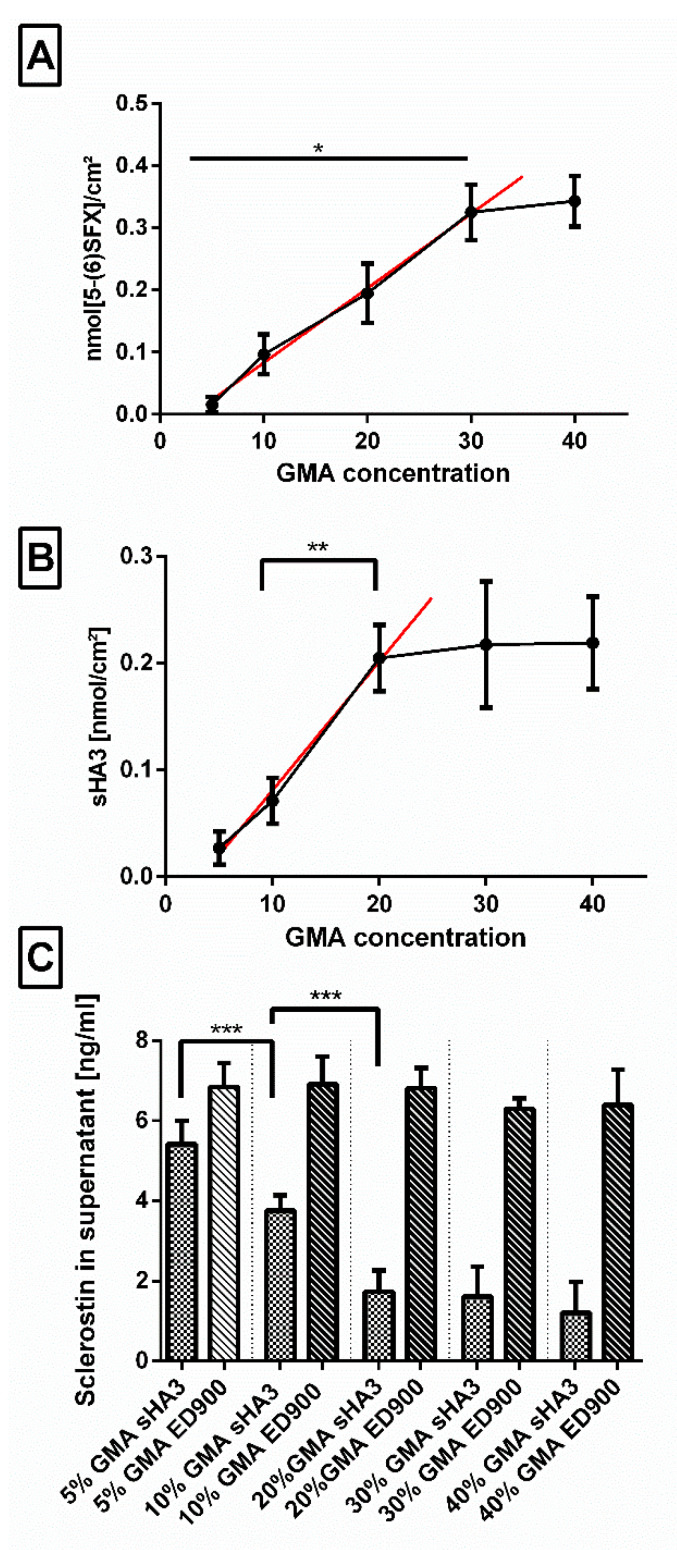
Effect of increasing concentrations (5–40% *m*/*m*) of GMA anchor incorporated during cross-copolymerization. Impact on (**A**) linker surface decoration, measured via 5-(6)SFX staining, (**B**) subsequent functionalization with fluorescent-labeled sHA3, and (**C**) scavenging capability of the sHA3 and ED900 control surfaces is shown. Film surfaces: ED900: ED900 incubated with soluble sHA3; sHA3: ED900 covalently coated with sHA3. Linear regression (**A**) *R*^2^ = 0.92, slope = 0.011, *y*-intercept = −0.037; (B) *R*^2^ = 0.90, slope = 0.012, *y*-intercept = −0.040. All values represent the mean ± SD of *n* ≥ 4. * *p* < 0.05; ** *p* < 0.01; *** *p* < 0.001 denotes significant difference vs. the respective formulations.

In order to find the most efficient material composition for effective scavenging, we varied the amount of GMA (5–40% *m*/*m*) in the film matrix and investigated decoration with linker (Figure 7A), sHA3 (Figure 7B), and sclerostin scavenging (Figure 7C).

In a range of 5–30%, we determined a continuous increase in available amino groups per surface area after incubation of the surfaces in the ED900 modification mixture which leveled off at higher GMA contents. Functionalization with sHA3, on the other hand, showed only increasing amounts up to 20% GMA. This was confirmed by an increase in the scavenging efficiency of our sHA3 surfaces in the same range.

### 3.3. Cell Culture

**Figure 8:**
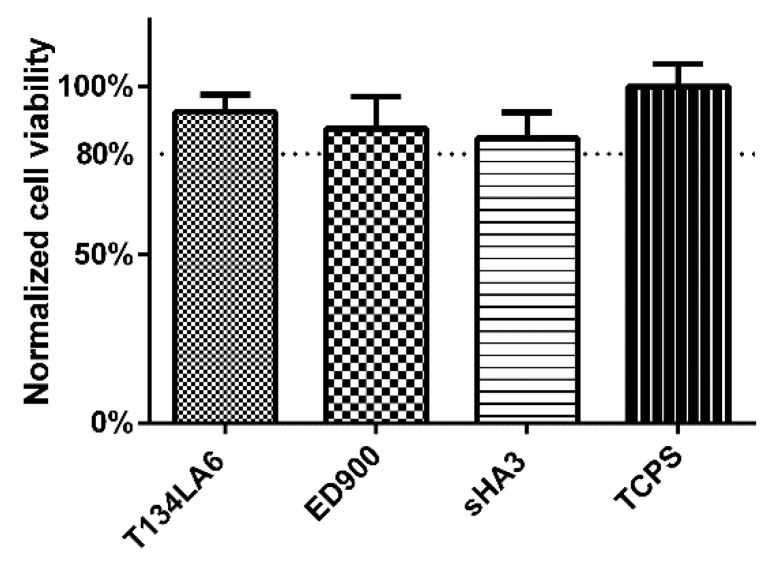
Direct-contact cytocompatibility of the different materials to L929 fibroblasts, normalized to TCPS control. Viability of the cells was determined after 24 h, using RotiTest^®^ Vital WST-8 assay. Film surfaces: T134LA6: unmodified macromer; ED900: surface coating with ED900 preincubated with soluble sHA3; sHA3: covalent ED900 and sHA3-coating; TCPS: tissue culture polystyrene. All values represent the mean ± SD of *n* ≥ 4.

We investigated cytocompatibility by direct culture of L929 fibroblasts on sHA3 and control surfaces. The WST-8 assay revealed a cell viability > 80% for all modifications of the films (Figure 8).

**Figure 9:**
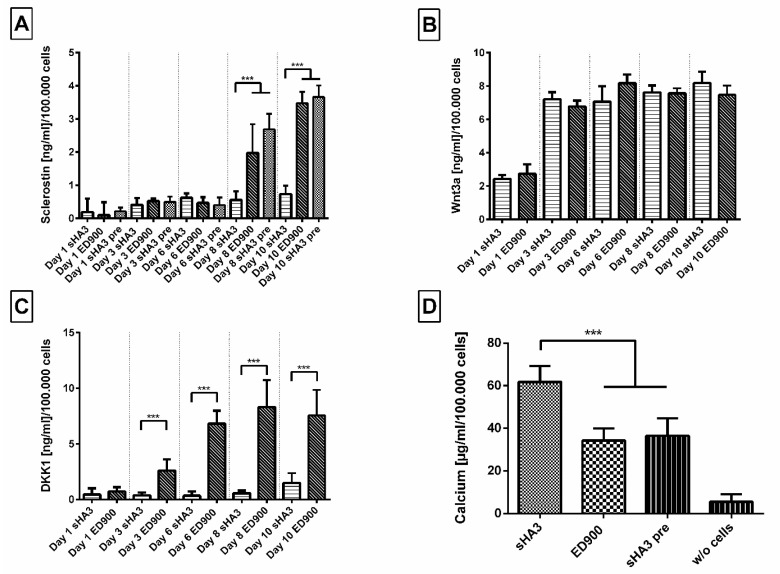
Effect of film modification on secreted protein content in the supernatant and mineralization during osteogenic differentiation of SaOS-2 cells. Cells were seeded on differently modified surfaces at a density of 10,000/cm^2^ and differentiated for 10 days. (**A**) Sclerostin, (**B**) Wnt3a, and (**C**) DKK1 were then quantified from the cell supernatant via ELISA after 1, 3, 6, 8, and 10 days. Calcium content (**D**) was quantified at day 10. Film surfaces: ED900: surface coating with ED900 preincubated with soluble sHA3; sHA3: covalent ED900 and sHA3-coating; sHA3 pre: sHA3 surface preincubated with 40 ng/mL sclerostin; *w*/*o* cells: sHA3 surface without seeded cells. All values represent the mean ± SD of *n* ≥ 4. *** *p* < 0.001 denotes significant difference vs. the respective surface modifications.

In a final step, we transferred our system to different cell culture systems in order to gain first insights in the relevance of our scavenging approach. In a culture period of 10 days, SaOS-2 cells undergoing osteogenic differentiation showed an increasing production of sclerostin starting on day 8 (Figure 9A). In the supernatant of cells seeded on the sHA3 functionalized films, we detected significantly lower amounts of sclerostin compared to ED900 control films. As a second control, covalently modified sHA3 films presaturated with sclerostin were tested for their potential impact and showed no significant differences when compared to ED900.

DKK1 as another relevant Wnt antagonist was found to increase on control surfaces starting on day 3. The amounts of DKK1 remained significantly lower in the supernatant of cells seeded on sHA3 films than on control films until the end of our experiment (Figure 9C). The quantification of Wnt3a in the supernatants, however, showed no significant differences between covalently sHA3 functionalized materials and the ED900 control (Figure 9B). As a marker for osteogenic differentiation, we determined the amount of calcium on day 10. Normalized calcium content of the sHA3 group was significantly higher in comparison to all controls (Figure 9D).

**Figure 10:**
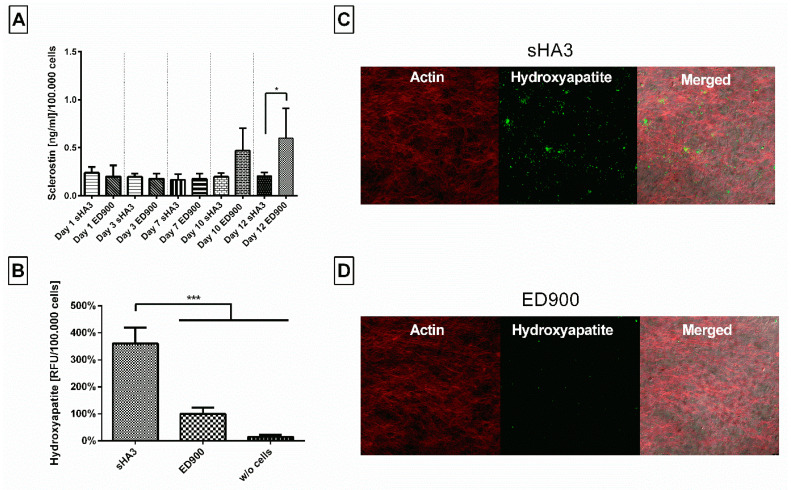
Effect of film modification on secreted protein content in the supernatant and mineralization during osteogenic differentiation of hMSC. Cells were seeded on differently modified surfaces at a density of 15,000/cm^2^ and differentiated for 12 days. (**A**) Sclerostin was then quantified from the cell supernatant via ELISA after 1, 3, 7, 10, and 12 days. OsteoImage^TM^ staining was assessed at day 12 and the fluorescence signal quantitatively (**B**) and qualitatively(**C**,**D**) analyzed. Film surfaces: ED900: surface coating with ED900 preincubated with soluble sHA3; sHA3: covalent ED900 and sHA3-coating; *w*/*o* cells: sHA3 surface without seeded cells. All values represent the mean ± SD of *n* ≥ 4. * *p* < 0.05; *** *p* < 0.001 denotes significant difference vs. the respective surface modifications.

In order to investigate the films with human primary cells, we differentiated hMSC on the film surfaces. Here, we determined significant differences in sclerostin in the supernatant of sHA3 and ED900 groups (Figure 10A) on day 12. Mineral deposition was chosen as a marker for osteogenic differentiation and detected with the hydroxyapatite specific OsteoImage^TM^ staining. Qualitative (Figure 10C,D) and quantitative (Figure 10B) assessment showed significantly higher fluorescent signal and thereby hydroxyapatite content on the sHA3 films.

## 4. Discussion

### 4.1. Design of the Material

According to our design concept, we generated films as model system for implant surfaces by cross-copolymerization of T134LA6 macromer with the small anchor molecule GMA. GMA as a small molecule provides high flexibility in the choice of solvent. Suitable solvents ideally keep all monomers dissolved and mobile during film polymerization and support homogeneous polymerization of the two components. Solvents were found to perform differently during film production [8,21]. Increasing viscosity due to strong interactions of the solvent with the macromer or fast evaporation during film formation may have caused inhomogeneous polymerization and eventually rough surfaces as observed in our study for ethyl acetate and THF. Solvent mixtures can be necessary if the monomers have different solubilities. Their application nevertheless may change composition in a thermal polymerization process and cause phase separation with increasing concentration [8]. Moreover, methacrylated monomers need to provide suitable reactivity for efficient copolymerization. A setup, which meets these demands, was successfully developed for the chosen combination of our biodegradable macromer and GMA. In the current study, dioxane provided smooth films with high availability of epoxy groups, which served as anchors for subsequent modification steps. We were able to use a single solvent for matrix synthesis and processed a solution of all components into a film by cross-copolymerization.

Subsequently, in an independent step, we decorated the film surface with polyetheramine linker molecules. Curing protocols involving 1–2 h at a high temperature of 80 °C were found ideal in our study. In principal, approaches involving the reaction of an epoxy group with amines have been applied in the context of biomaterials for different applications, e.g., to modify GMA polyHIPE monoliths [22,23] and epoxy composites [24]. In addition, the necessity of high temperatures to enable sufficient conversion rates of GMA with various amines has been shown in literature [23,25]. More work has been published on the formation of nanoparticles made of bisepoxide and polyetheramines [26,27].

#### 4.1.1. Impact of Linker Properties on Surface Decoration

It is well known that length and accessibility of the linker is a key point in successful functionalization of biomaterial surfaces [28] and nanoparticles [6]. In this context, the influence of systematically varied size and valence of the linker molecules on the epoxy conversion was determined by the amount of free amino groups available on the surface as a measure for grafted polyetheramines.

In general, we found a correlation of the molecular weight of polyetheramines and the efficiency of surface decoration, resulting in denser modifications for smaller molecules. EDR148, the smallest polyetheramine investigated in this study, reacted fast and resulted in the highest surface decoration density. With larger molecular weight, diffusion coefficient decreases and steric hindrance after immobilization may inhibit further modification of the surface. Low differences were found between ED600 and ED900, whereas ED2003 showed strongly reduced immobilized amounts on the film surfaces. Incubation with both three-armed polyetheramines (T403 and T3000), resulted in a relatively low number of free amino groups available on the surface. In other publications, a higher initial activation energy for three armed polyetheramines in comparison to a two armed equivalent was found [29]. For the T3000, its high molecular weight, resulting in a low number of amino groups per molecular weight in combination with a low reactivity may be a reason leading to lowest of all measured conversion rates.

When the conditions for linker binding were applied for more than 1 h on the EDR148 or more than 2 h on the ED600 and ED900 incubation, we detected increasing polyetheramine linkage even on GMA-free control films. We assume that linker binding under these conditions resulted from temperature driven amid formation with oligolactide esters of the biodegradable film material [19,30,31].

#### 4.1.2. Availability of Linker Molecules for sHA3 Functionalization

Subsequently biologically active sHA3-functionalization of T134LA6/GMA films decorated with EDR148, ED600, and ED900 was conducted. Sulfated glycosaminoglycans were chosen here as they are known to beneficially influence bone regeneration. Mechanisms postulated by literature are the support of osteoblast functions [11] and the regulation of Wnt signaling pathways [13,32].

Covalent binding of sHA3 to the polyetheramine decorated surfaces was achieved by established EDC/NHS chemistry. In absence of the coupling reagent, however, we found strong adsorptive interaction between amino-modified control surfaces and sHA3. We generated surfaces carrying acetylated groups in order to demonstrate the actual impact of our cationic polyetheramine surfaces on these ionic interactions. Very low adsorptive binding took place on these control surfaces. In contrast to established washing buffers (HEPES, TRIS) and water, a thiocyanate buffer was found to be able to remove the negatively charged sHA3 molecules from the cationic ED900 control films.

Quantitative comparison of the sHA3 functionalization of EDR148, ED600, and ED900 linker decorated materials, however, revealed decisive differences. The low efficiency of covalent sHA3 surface decoration on the EDR148 modified materials leads to the conclusion, that this linker, regardless of its reactivity, is too short to allow a sufficient covalent binding of the relatively large sHA3 and thereby functionalization. In addition, we found particular strong adsorptive interaction between the densely linker decorated films and sHA3 that was not removable even with the thiocyanate buffer. These ionic interactions may have reduced the availability of sHA3 for covalent binding.

With increasing molecular weight of the polyetheramines, we assume that the availability for covalent binding increases while ionic interactions decrease as can be seen by the decreasing amounts of sHA3 on the respective control films. All the measured properties led to the conclusion to use ED900 as the most suitable linker, combining good modification properties with the best ability to react with sHA3 and thereby functionalize the surface.

Overall the post-polymerization modification approach provides an opportunity to apply different kinds of linkers or even direct functionalization of the material as stated in literature [33]. Nevertheless, our own as well as other studies suggest the necessity to use a spacer in order to effectively bind bioactive molecules to functionalize surfaces [34,35].

#### 4.1.3. Influence of the Concentration of Anchor Molecule Applied during Polymerization

When we determined the optimal amount of the GMA anchor applied during cross-copolymerization, we found a linear correlation between the GMA content in the films and ED900 immobilization between 5% and 30% GMA. sHA3 functionalization of the ED900 decorated surfaces again correlated with the GMA content, however, with the large sHA3 the linear range was limited to GMA contents of 20% as expected by increasing steric hindrance.

Overall, we showed the ability of the presented system to adapt linker and consequently functionalization density in an accessible manner. Possible applications besides customized functionalization regarding the materials intended purpose, could be a precise control over surface properties, e.g., cell adhesion or hydrophilicity [36,37].

#### 4.1.4. Adjusted Conditions for Optimized sHA3 Functionalization

The optimal properties of a linker applied in this specific scenario seem to be on the one hand a certain degree of reactivity, shown for smaller linkers and on the other hand a sufficient accessibility allowing for a subsequent functionalization via EDC/NHS chemistry. Depending on whether a covalent or adsorptive binding is desirable for future applications, variations of the linker are easily possible (ED900 vs EDR148). It is conceivable that a functionalization with smaller molecules may work with EDR148, as the amino groups were available for a smaller molecule, namely the dye 5-(6)SFX.

For the purpose of functionalization with sHA3, the immobilization of ED900 as a linker resulted in the most promising characteristics. When the envisaged application requires a longer linker polyetheramine, ED2003 may be a promising candidate.

The concentration of GMA in the final formulation was chosen as 20% (*m*/*m*) as a further increase of amino groups did not enhance the ability to functionalize the material. All in all, an anchor concentration in this range assures a high enough reaction capacity [8,13] while still keeping processible T134LA6 properties.

L929 fibroblasts, as recommended by guidelines for cytotoxicity studies of polymers [38], were chosen. After direct contact with the respective material, a WST-8 cytotoxicity assay revealed good cell viability on the basic and linker modified material as well as functionalized surfaces. Lower cell numbers on the sHA3 films that have been shown in a cell adhesion study before [13] may explain the slightly lower turnover of the WST-8 reagent.

Overall, our system enables a straightforward adaption of linker length and density, properties that have been shown to be of great importance regarding cell–material interaction [6].

### 4.2. Scavenging of Wnt Antagonists

In order to reduce the local sclerostin concentrations, we intended to modify implant surfaces with sHA3. The inactivation of sclerostin has been shown to have a positive impact on bone formation [39]. The application of sclerostin-binding antibody romosozumab increased bone mineral density as well as bone formation and resulted in decreased bone resorption in osteoporosis patients [40,41]. While the application of antibodies showed great efficiency, their systemic administration is often accompanied with side effects [42]. The approach presented here would allow for a selective scavenging at the defect site via sHA3 functionalization of either functionalized T134LA6 scaffolds or thin films polymerized onto established biomaterials.

Strong interactions of sclerostin and sulfated glycosaminoglycans in general have been shown before [14]. We confirmed the sulfation- and concentration dependent affinity of our sHA3 towards sclerostin by SPR interaction analysis. [14].

In a next step, we transferred these findings to our model film system and showed a significant reduction of detectable sclerostin in supernatants in contact with sHA3 surfaces, indicating a successful scavenging of the protein. The overall scavenging capacity of the films resulted in values of 11.5 ng/mL per cm^2^. Studies reported elevated concentrations of 0.685 ng/mL in the serum of osteoporosis patients [16] and up to 1.75 ng/mL in women with osteoporotic fractures [43]. This indicates that a local scavenging effect of our sHA3 functionalized materials may be relevant in vivo. The observed controllability of functionalization density via the amount of anchor molecules correlated with scavenging capacities.

Sclerostin, a Wnt antagonist, is part of a complex system including Wnt3a as an important agonist. Binding of Wnt3a to sHA3-functionalized films supported a previous finding for the interaction of molecules carrying a heparin-binding domain [44]. In a competitive incubatory approach, we showed, however, that sclerostin and DKK1 are the preferred binding partners of sHA3 over Wnt3a. In addition, coincubation with an equal molar amount of DKK1 and Wnt3a did not interfere with the scavenging of sclerostin.

In order to investigate biological effects in vitro, we cultured the fast differentiating SaOS-2 cell line as well as hMSC on sHA3-modified and control films. Both cell types secreted DKK-1 and Wnt3a [13] earlier than sclerostin. Wnt3a levels remained in the culture medium at control levels, whereas DKK-1 and sclerostin were effectively scavenged by the sHA3-decorated films, confirming the results of the coincubation. In addition to the previously shown scavenging of DKK1 up to day 6 [13], now a scavenging effect over 10 days was demonstrated. We tested for calcium accumulation as a first measure of osteogenic differentiation. The cultivation on sHA3 films resulted in a higher mineralization of cells as compared to controls.

For hMSC undergoing osteogenic differentiation, we were able to prove the binding of sclerostin in this setting, in addition to the published scavenging of DKK1 [13]. However, sclerostin levels increased not before day 12. At this time point, we determined efficient scavenging by sHA3 decorated films. Quantification of hydroxyapatite as a marker for the status of osteogenic differentiation revealed an increased deposition on sHA3 films. In comparison to earlier studies [13], which focused on the matrix mineralization at earlier time points for both cell types and thereby solely on the impact of scavenging DKK1, this study showed that the effect on osteogenic differentiation may result from scavenging of both Wnt antagonists. A beneficial effect of the combined inactivation of both Wnt antagonists, potentially resulting in synergistic osteoanabolic effects has been discussed before [45]. In previously published studies mineralization correlated well with later time points and even an improved in-vivo fracture healing [9,11].

### 4.3. Limitations

Beside the discussed analytical benefits of our system, we recognize some potential limitations. We focused on establishing a platform allowing for multiple variations and decided to show its applicability in a distinct scenario. Future studies may reveal a diverse range of applications via multiple functionalization options utilizing amino groups [5]. The successful scavenging of sclerostin and DKK1 by sHA3 functionalized macromer surfaces has been shown in a cell culture system and needs to be evaluated further and in an in-vivo approach. Those studies may provide additional insights in the processes underlying osteogenic differentiation and its markers.

## 5. Conclusions

In this study, we showed that cross-copolymerized materials from a biodegradable macromer and GMA allowed for precise control of surface properties of model films. Epoxy-groups were well available for polyetheramine decoration and subsequent functionalization with pharmaceutically active sHA3. The film platform allows for systematic investigation and characterization with sophisticated methods available only for flat surfaces.

Dividing the process of polymerization, linker-modification, and functionalization into three separated steps, permits variations of each of them independently. We successfully immobilized different polyetheramine linkers and varied the linker density on the surface. The shown accessibility of the amino groups enables variations of the functionalizing pharmaceutically active molecule in future studies. 

Covalent functionalization with sHA3 provides high scavenging efficiency for Wnt antagonists DKK-1 and sclerostin. The ability of our functionalized films to scavenge sclerostin and concomitantly DKK1 from medium as well as in a cell culture environment was shown and holds promise for the development of therapeutic applications of this material.

## Supplementary Materials

The following are available online at https://www.mdpi.com/1999-4923/12/11/1037/s1, Figure S1: Illustration of the material composition.

## Abbreviations

5(6)-SFX: 6-(fluorescein-5-(and-6)-carboxamido) hexanoic acid succinimidyl ester, DKK1: Dickkopf-1, DMF: N, N-dimethylformamide, DMP30: 2, 4, 6-tris(dimethylaminomethyl)phenol, EDC: 1-ethyl-3-(-3-dimethylaminopropyl) carbodiimide hydrochloride, FBS: fetal bovine serum, GAG: glycosaminoglycan, GMA: glycidyl methacrylate, HA: hyaluronan, HEPES: 4-(2-hydroxyethyl)-1-piperazineethanesulfonic acid, hMSC: human mesenchymal stem cells, MW: molecular weight, NHS: N-hydroxysuccinimide, PBS: phosphate-buffered saline, SD: standard deviation, sHA3: high-sulfated hyaluronan, SOST: sclerostin, TCPS: tissue culture polystyrene, THF: tetrahydrofuran, TRIS: tris(hydroxymethyl)-aminomethane, Wnt: wingless-related integration site.
