# Supplementary material for: A Versatile Macromer-Based Glycosaminoglycan (sHA3) Decorated Biomaterial for Pro-Osteogenic Scavenging of Wnt Antagonists
Source: Pharmaceutics. 2020 Oct 29;12(11):1037. doi: 10.3390/pharmaceutics12111037 (PMC7693161; doi:10.3390/pharmaceutics12111037)
Supplement: Supplementary file 1 [file pharmaceutics-12-01037-s001.pdf]

Supplementary Materials: Versatile Macromer-Based Glycosaminoglycan (sHA3) Decorated Biomaterial for Pro-Osteogenic Scavenging of Wnt Antagonists

Mathis Gronbach, Franziska Mitrach, Stephanie Möller, Sandra Rother, Sabrina Friebe, Stefan G. Mayr, Matthias Schnabelrauch, Vera Hintze, Michael C. Hacker and Michaela Schulz-Siegmund

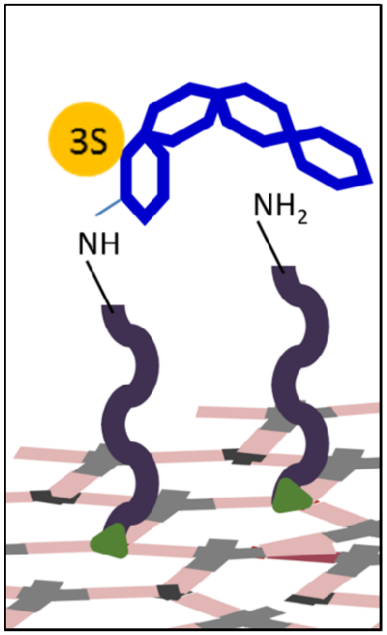

Symbol	Name	Function	Structure
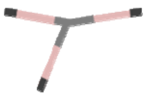	T134LA6	Basic macromer	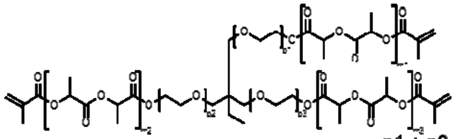 p1 + p2 + p3 = 0
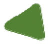	GMA	Anchor	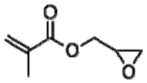
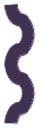	ED900	Linker	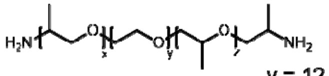 y = 12.5; (x+z) = 6.0
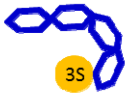	sHA3	Functionalization	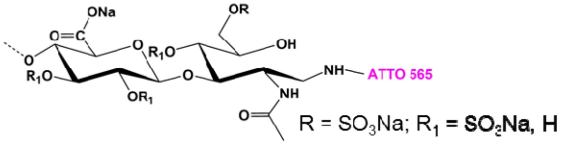

Figure S1. Illustration of the material composition. Left: Schematic section of the functionalized film. Right: Table providing name, function and structure of the functionalized films.
